# Early Estimates of Bivalent mRNA Booster Dose Vaccine Effectiveness in Preventing Symptomatic SARS-CoV-2 Infection Attributable to Omicron BA.5– and XBB/XBB.1.5–Related Sublineages Among Immunocompetent Adults — Increasing Community Access to Testing Program, United States, December 2022–January 2023

**DOI:** 10.15585/mmwr.mm7205e1

**Published:** 2023-02-03

**Authors:** Ruth Link-Gelles, Allison Avrich Ciesla, Lauren E. Roper, Heather M. Scobie, Akilah R. Ali, Joseph D. Miller, Ryan E. Wiegand, Emma K. Accorsi, Jennifer R. Verani, Nong Shang, Gordana Derado, Amadea Britton, Zachary R. Smith, Katherine E. Fleming-Dutra

**Affiliations:** ^1^National Center for Immunization and Respiratory Diseases, CDC; ^2^Eagle Health Analytics, San Antonio, Texas; ^3^Center for Preparedness and Response, CDC. ^4^Epidemic Intelligence Service, CDC.

The SARS-CoV-2 Omicron sublineage XBB was first detected in the United States in August 2022.* XBB together with a sublineage, XBB.1.5, accounted for >50% of sequenced lineages in the Northeast by December 31, 2022, and 52% of sequenced lineages nationwide as of January 21, 2023. COVID-19 vaccine effectiveness (VE) can vary by SARS-CoV-2 variant; reduced VE has been observed against some variants, although this is dependent on the health outcome of interest. The goal of the U.S. COVID-19 vaccination program is to prevent severe disease, including hospitalization and death (*1*); however, VE against symptomatic infection can provide useful insight into vaccine protection against emerging variants in advance of VE estimates against more severe disease. Data from the Increasing Community Access to Testing (ICATT) national pharmacy program for SARS-CoV-2 testing were analyzed to estimate VE of updated (bivalent) mRNA COVID-19 vaccines against symptomatic infection caused by BA.5-related and XBB/XBB.1.5-related sublineages among immunocompetent adults during December 1, 2022–January 13, 2023. Reduction or failure of spike gene (*S*-gene) amplification (SGTF) in real-time reverse transcription–polymerase chain reaction (RT-PCR) was used as a proxy indicator of infection with likely BA.5-related sublineages and *S*-gene target presence (SGTP) of infection with likely XBB/XBB.1.5-related sublineages (*2*). Among 29,175 nucleic acid amplification tests (NAATs) with SGTF or SGTP results available from adults who had previously received 2–4 monovalent COVID-19 vaccine doses, the relative VE of a bivalent booster dose given 2–3 months earlier compared with no bivalent booster in persons aged 18–49 years was 52% against symptomatic BA.5 infection and 48% against symptomatic XBB/XBB.1.5 infection. As new SARS-CoV-2 variants emerge, continued vaccine effectiveness monitoring is important. Bivalent vaccines appear to provide additional protection against symptomatic BA.5-related sublineage and XBB/XBB.1.5-related sublineage infections in persons who had previously received 2, 3, or 4 monovalent vaccine doses. All persons should stay up to date with recommended COVID-19 vaccines, including receiving a bivalent booster dose when they are eligible.

ICATT is designed to increase access to SARS-CoV-2 testing in areas with high social vulnerability^†^ through testing at selected pharmacy- and community-based testing sites nationwide.^§^ ICATT VE methods have been described previously (*3*,*4*). Briefly, at test registration, adults report information on vaccination history,^¶^ current COVID-19–like illness symptoms, previous positive SARS-CoV-2 test results, and underlying medical conditions. Adults receiving testing at participating sites during December 1, 2022–January 13, 2023, who reported one or more COVID-19–like illness symptoms were included. For this analysis, eligible tests were those performed at a commercial laboratory that used the real-time RT-PCR TaqPath COVID-19 Combo Kit (ThermoFischer Scientific); quantitative results were reported as cycle threshold (Ct) values for each of three SARS-CoV-2 gene targets (*S*, *N*, and *ORF1ab*). Specimens with missing Ct values for *N* or *ORF1ab* were excluded. SARS-CoV-2–positive specimens with either null or reduced amplification of the spike *S*-gene (Ct for *S-*gene >4 cycles from the average of *N* and *ORF1ab* Ct values) were considered to have SGTF (*5*); SARS-CoV-2–positive specimens without SGTF were considered to exhibit SGTP. SGTF or SGTP can serve as a proxy marker of SARS-CoV-2 lineages and sublineages with or without a deletion of amino acids 69–70 in the N-terminal domain of the spike protein, respectively (*5*). Currently circulating SARS-CoV-2 variants were classified by SGTF (BQ.1.1, BQ.1, BF.7, and other BA.4 and BA.5 sublineages) and SGTP (XBB.1.5, XBB, BN.1, and other BA.2 sublineages). During the week ending December 3, 2022, approximately 13% of specimens sequenced nationwide were BA.2 sublineages, including 2.4% XBB.1.5 (95% CI = 0.6%–6.2%) and 5.0% XBB (95% CI = 3.7%–6.6%); by the end of the analytic period, these proportions had risen to approximately 41%, including 37.2% XBB.1.5 (95% prediction interval [PI] = 26.8%–49.0%) and 4.0% XBB (95% PI = 3.3%–4.7%).**

Case-patients were persons who received a positive laboratory-based NAAT result classified as SGTF (BA.5-related) or SGTP (XBB/XBB.1.5-related); control-patients were those who received a negative NAAT result. Tests among persons fulfilling any of following criteria were excluded from analyses: 1) presence of an immunocompromising condition^††^; 2) unvaccinated or receipt of only 1 COVID-19 vaccine dose; 3) receipt of a non-mRNA COVID-19 vaccine; 4) receipt of >4 monovalent mRNA doses if aged ≥50 years or >3 monovalent doses if aged 18–49 years; or 5) receipt of only 2 mRNA doses, with the second dose received <4 months before the SARS-CoV-2 test. Persons reporting an mRNA booster dose on or after September 1, 2022, were assumed to have received a bivalent dose because monovalent mRNA doses were not authorized for use as booster doses at that time.^§§^ In addition, tests from persons who reported a positive SARS-CoV-2 test result during the preceding 90 days^¶¶^ were excluded to avoid analyzing multiple tests for the same illness episode or reinfections within a relatively short time frame. Relative VE of a bivalent booster dose was calculated by comparing odds of receipt of a bivalent booster dose with those of no bivalent booster dose among persons who had received 2–4 monovalent vaccine doses. Odds ratios (ORs) were estimated using multivariable logistic regression***; VE was calculated separately based on SGTF/SGTP status as (1 − OR) x 100. 

As of January 16, 2023, genomic sequencing data were available for a random subset of ICATT specimens with SGTP and collection dates through January 2, showing an increase in XBB.1.5 prevalence over time. During December 1, 2022–January 2, 2023, XBB.1.5 comprised 33% (495) of specimens exhibiting SGTP. During the interval December 11–January 2, XBB.1.5 accounted for 38% of sequenced ICATT specimens with SGTP (377), and during the interval December 18–January 2, XBB.1.5 accounted for 43% of sequenced ICATT specimens with SGTP (252)(*2*). As XBB.1.5 has continued to increase nationwide, true proportions of XBB.1.5 in the analytic dataset, which included tests through January 13, were likely higher, but sequencing results were not yet available for specimens collected during the whole period. Sensitivity analyses were conducted using two intervals, December 11, 2022–January 13, 2023, and December 18, 2022–January 13, 2023, to assess the effect of different proportions of the XBB.1.5 sublineage among SGTP cases during early December. Analyses were conducted using R software (version 4.1.2; R Foundation). This activity was reviewed by CDC and was conducted consistent with applicable federal law and CDC policy.^†††^

Among 29,175 NAAT results among persons with COVID-19–like illness symptoms eligible for this analysis, 13,648 (47%) were positive for SARS-CoV-2, including 10,596 (78%) with SGTF (BA.5-related) and 3,052 (22%) with SGTP (XBB/XBB.1.5-related) (Table 1). More control-patients who received negative SARS-CoV-2 test results reported having received a bivalent COVID-19 mRNA booster (34%) than did case-patients with positive SARS-CoV-2 test results (SGTF = 22%; SGTP = 21%). Among those who had received only monovalent vaccine doses, 45% reported a positive SARS-CoV-2 test result >90 days before the current test, compared with 34% among those who received a bivalent dose. Among those who had received only monovalent vaccine doses, the median interval since the last dose was 13 months (IQR = 11–17) for case-patients and 13 months (IQR = 11–18) for control-patients.

**TABLE 1:** Characteristics of patients with SARS-CoV-2 nucleic acid amplification tests, by* S*-gene target status (N = 29,175) — Increasing Community Access to Testing program, United States, December 1, 2022–January 13, 2023

Characteristic	No. (Col. %)		
	SARS-CoV-2 control-patients with negative test results	SARS-CoV-2 case-patients with positive test results	
		SGTF (likely BA.5-related)	SGTP (likely XBB/ XBB.1.5-related)
**All tests**	**15,527**	**10,596**	**3,052**
**Age group, yrs**			
18–49	9,907 (64)	6,353 (60)	1,860 (61)
50–64	3,218 (21)	2,639 (25)	784 (26)
≥65	2,402 (15)	1,604 (15)	408 (13)
**Gender**			
Female	9,951 (64)	6,214 (59)	1,771 (58)
Male	5,495 (35)	4,347 (41)	1,269 (42)
Other	81 (1)	35 (0.3)	12 (0.4)
**Race and ethnicity**			
Black or African American, NH	1,897 (12)	1,216 (11)	417 (14)
Hispanic or Latino	3,047 (20)	2,558 (24)	742 (24)
White, NH	7,576 (49)	4,629 (44)	1,260 (41)
Other, NH	2,100 (14)	1,539 (15)	433 (14)
Unknown	907 (6)	654 (6)	200 (7)
HHS testing site region*			
Region 1	1,280 (8)	583 (6)	383 (13)
Region 2 – New York and New Jersey only	1,498 (10)	870 (8)	693 (23)
Region 2 – Puerto Rico only	107 (1)	115 (1)	43 (1)
Region 3	913 (6)	538 (5)	232 (8)
Region 4	3,043 (20)	1,899 (18)	535 (18)
Region 5	3,330 (21)	2,355 (22)	412 (13)
Region 6	1,738 (11)	1,168 (11)	254 (8)
Region 7	326 (2)	204 (2)	35 (1)
Region 8	400 (3)	228 (2)	45 (1)
Region 9	2,673 (17)	2,524 (24)	410 (13)
Region 10	219 (1)	112 (1)	10 (0.3)
**SVI, mean (SD)^†^**	0.5 (0.3)	0.5 (0.3)	0.5 (0.3)
**History of self-reported SARS-CoV-2 positive test result**			
None	7,556 (49)	7,546 (71)	1,921 (63)
Positive >90 days before current test	7,971 (51)	3,050 (29)	1,131 (37)
**Self-reported ≥1 chronic underlying condition** ^§^			
No	9,376 (60)	6,323 (60)	1,899 (62)
Yes	6,151 (40)	4,273 (40)	1,153 (38)
**Among persons who received only monovalent mRNA doses, no. of monovalent doses** ^¶^			
2	5,131 (50)	3,933 (47)	1,125 (47)
3	4,415 (43)	3,754 (45)	1,111 (46)
4**	692 (7)	594 (7)	162 (7)
**Received bivalent booster dose**			
No	10,238 (66)	8,281 (78)	2,398 (79)
Yes	5,289 (34)	2,315 (22)	654 (21)
**Among persons who received a bivalent mRNA dose, no. of monovalent doses**			
2	580 (11)	248 (11)	69 (11)
3	3,427 (65)	1,433 (62)	427 (65)
4**	1,282 (24)	634 (27)	158 (24)

**Abbreviations:** HHS = U.S. Department of Health and Human Services; ICATT = Increasing Community Access to Testing program; NH = non-Hispanic; SGTF = *S*-gene target failure; SGTP = *S*-gene target presence; SVI = Social Vulnerability Index.

* Regions are defined by HHS and include only states and territories with ICATT sites. U.S. Virgin Islands (Region 2) and American Samoa, Federated States of Micronesia, Guam, Marshall Islands, Northern Mariana Islands, and Palau (Region 9) were not included because they did not have pharmacies participating in ICATT. States included in each region are available at https://www.hhs.gov/about/agencies/iea/regional-offices/index.html. For purposes of this analysis and because of the regional pattern of XBB-lineage infections, HHS Region 2 was split into “Region 2 – New York and New Jersey only” and “Region 2 – Puerto Rico only.”

^†^ SVI is a tool that uses U.S. Census Bureau data on 16 social factors to rank social vulnerability by U.S. Census Bureau tract. The scale is from zero to 1; higher SVIs represent more vulnerable communities. Tests with missing SVI data (<1% of total) were excluded from all analyses. https://www.atsdr.cdc.gov/placeandhealth/svi/data_documentation_download.html

^§^ Underlying conditions included on the test registration questionnaire were heart conditions, high blood pressure, overweight or obesity, diabetes, current or former smoker, kidney failure or end stage renal disease, cirrhosis of the liver, and chronic lung disease (e.g., chronic obstructive pulmonary disease, moderate to severe asthma, cystic fibrosis, or pulmonary embolism).

^¶^ Test registrants who reported receiving COVID-19 vaccines were asked to report the total number of doses and manufacturers of vaccines received and for the most recent dose, month, and year of receipt; therefore, the number of months between a vaccine dose and testing is a whole number calculated as the difference between the month and year of testing and the month and year of the vaccine dose. Persons reporting an mRNA booster dose on or after September 1, 2022, were assumed to have received a bivalent dose because no monovalent mRNA doses were authorized for use as booster doses at that time. For doses received in the same month or the month before SARS-CoV-2 testing, an additional question was asked to specify whether the dose was received ≥2 weeks before testing, and only doses received ≥2 weeks before testing were included.

** Persons aged <50 years without moderate or severe immunocompromise were not eligible for a fourth monovalent (second booster) dose.

Across age groups, VE was generally similar against BA.5-related infections and XBB/XBB.1.5-related infections. VE against symptomatic BA.5-related infection was 52% among persons aged 18–49 years, 43% among persons aged 50–64, and 37% among those aged ≥65 years (Table 2). VE against symptomatic XBB/XBB.1.5-related infection was 49% among persons aged 18–49, 40% among persons aged 50–64 years, and 43% among those aged ≥65 years. Evidence of waning VE by 2–3 months after receiving a bivalent dose based on point estimates was minimal, although estimates were imprecise. Sensitivity analyses did not show a meaningful change in VE by different analytic period start dates (Table 3).

**TABLE 2:** Relative vaccine effectiveness* of a single bivalent mRNA COVID-19 booster received after 2–4 monovalent vaccine doses against symptomatic SARS-CoV-2 infection, by age group and* S*-gene target status — Increasing Community Access to Testing program, United States, December 1, 2022–January 13, 2023

Age group, yrs/mRNA dosage pattern^†^	Total no of tests	SARS-CoV-2 negative test results	SARS-CoV-2–positive test results by *S*-gene target status			
			SGTF (likely BA.5-related)		SGTP (likely XBB/XBB.1.5-related)	
		No. (row %)	No. (row %)	VE (95% CI)	No. (row %)	VE (95% CI)
**18–49**						
Received 2–3 monovalent doses only (Ref)^§^	13,921	7,043 (51)	5,326 (38)	—	1,552 (11)	—
Overall (≥2 weeks since bivalent booster dose)	4,199	2,864 (68)	1,027 (24)	52 (48–56)	308 (7)	49 (41–55)
0–1 month since bivalent booster	1,056	716 (68)	262 (25)	51 (43–58)	78 (7)	50 (36–61)
2–3 months since bivalent booster	3,143	2,148 (68)	765 (24)	52 (48–56)	230 (7)	48 (39–55)
**50–64**						
Received 2–4 monovalent doses only (Ref)	4,603	2,036 (44)	1,983 (43)	—	584 (13)	—
Overall (≥2 weeks since bivalent booster dose)	2,038	1,182 (58)	656 (32)	43 (36–49)	200 (10)	40 (28–50)
0–1 month since bivalent booster	538	336 (62)	149 (28)	54 (43–63)	53 (10)	45 (25–60)
2–3 months since bivalent booster	1,500	846 (56)	507 (34)	39 (30–46)	147 (10)	38 (24–50)
**≥65**						
Received 2–4 monovalent doses only (Ref)	2,393	1,159 (48)	972 (41)	—	262 (11)	—
Overall (≥2 weeks since bivalent booster dose)	2,021	1,243 (62)	632 (31)	37 (28–44)	146 (7)	43 (29–55)
0–1 month since bivalent booster	381	260 (68)	94 (25)	55 (42–65)	27 (7)	50 (24–68)
2–3 months since bivalent booster	1,640	983 (60)	538 (33)	32 (21–40)	119 (7)	42 (26–54)

**Abbreviations**: Ref = referent group; SGTF = *S*-gene target failure; SGTP = *S*-gene target presence; VE = vaccine effectiveness.

* VE = (1 – adjusted odds ratio) x 100. Odds ratios were calculated using multivariable logistic regression, adjusting for single year of age, gender, race, ethnicity, Social Vulnerability Index of the testing location (<0.5 versus ≥0.5), underlying conditions (presence versus absence), U.S. Department of Health and Human Services region, local incidence (cases per 100,000 by individual county and state in the 7 days before test date), and testing calendar date.

^†^ For doses received in the same month or the month preceding SARS-CoV-2 testing, an additional question was asked to specify whether the dose was received ≥2 weeks before testing, and only doses received ≥2 weeks before testing were included.

^§^ Persons aged <50 years without moderate or severe immunocompromise were not eligible for a fourth monovalent (second booster) dose, so the Ref for this age stratum includes only those who received 2–3 monovalent doses.

**TABLE 3:** Sensitivity analyses of relative vaccine effectiveness* of a single bivalent mRNA COVID-19 booster dose received after 2–4 monovalent vaccine doses against symptomatic SARS-CoV-2 XBB/XBB.1.5 infection, by age group during two time intervals — Increasing Community Access to Testing program, United States, December 11, 2022–January 13, 2023, and December 18, 2022–January 13, 2023

Age group, yrs/Interval/mRNA dosage pattern^†^	Total no. of tests^§^	SARS-CoV-2–negative test results	SARS-CoV-2 positive test results with SGTP (likely XBB/XBB.1.5-related)	
		No. (row %)	No. (row %)	VE (95% CI)
**18–49**				
Dec 11, 2022–Jan 13, 2023				
Received 2–3 monovalent doses only (Ref)^¶^	10,335	5,213 (50)	1,334 (13)	—
Overall (≥2 weeks since bivalent booster dose)	3,262	2,216 (68)	261 (8)	51 (43–58)
Dec 18, 2022–Jan 13, 2023				
Received 2–3 monovalent doses only (Ref)	7,901	4,005 (51)	1,129 (14)	—
Overall (≥2 weeks since bivalent booster dose)	2,503	1,709 (68)	218 (9)	51 (42–58)
**50–64**				
Dec 11, 2022–Jan 13, 2023				
Received 2–4 monovalent doses only (Ref)	3,433	1,494 (44)	505 (15)	—
Overall (≥2 weeks since bivalent booster dose)	1,555	890 (57)	177 (11)	40 (26–51)
Dec 18, 2022–Jan 13, 2023				
Received 2–4 monovalent doses only (Ref)	2,612	1,144 (44)	428 (16)	—
Overall (≥2 weeks since bivalent booster dose)	1,209	697 (58)	146 (12)	42 (27–53)
**≥65**				
Dec 11, 2022–Jan 13, 2023				
Received 2–4 monovalent doses only (Ref)	1,839	903 (49)	227 (12)	—
Overall (≥2 weeks since bivalent booster dose)	1,547	940 (61)	128 (8)	42 (26–55)
Dec 18, 2022–Jan 13, 2023				
Received 2–4 monovalent doses only (Ref)	1,441	708 (49)	194 (13)	—
Overall (≥2 weeks since bivalent booster dose)	1,204	732 (61)	111 (9)	41 (23–55)

**Abbreviations:** Ref = referent group; SGTF = *S*-gene target failure; SGTP = *S*-gene target presence; VE = vaccine effectiveness.

* VE = (1 – adjusted odds ratio) x 100. Odds ratios were calculated using multivariable logistic regression, adjusting for single year of age, gender, race, ethnicity, Social Vulnerability Index of the testing location (<0.5 versus ≥0.5), underlying conditions (presence versus absence), U.S. Department of Health and Human Services region, local incidence (cases per 100,000 by individual county and state in the 7 days before test date), and testing calendar date.

^†^ For doses received in the same month or the month before SARS-CoV-2 testing, an additional question was asked to specify whether the dose was received ≥2 weeks before testing; only doses received ≥2 weeks before testing were included.

^§^ Total tests include those that returned positive results for SARS-CoV-2 and had SGTF but are not included in this table. Row totals do not sum to 100%.

^¶^ Persons aged <50 years without moderate or severe immunocompromise were not eligible for a fourth monovalent (second booster) dose, so the Ref for this age strata includes only those who received 2–3 monovalent doses.

## Discussion

This report provides the first estimates of bivalent mRNA COVID-19 VE against symptomatic SARS-CoV-2 infection with XBB-related sublineages. These preliminary estimates from national pharmacy testing conducted during December 1, 2022–January 13, 2023, showed relative bivalent booster dose VE (compared with 2–4 monovalent doses) to be similar for XBB/XBB.1.5 sublineage–related infections and BA.5 sublineage–related infections. VE estimates for both sublineages included in this analysis were similar to estimates from the same ICATT network published during a period of Omicron BA.5, BQ.1, and BQ.1.1 sublineage circulation in fall 2022 (*6*).

Early immunogenicity studies indicating lower neutralizing activity against XBB compared with other Omicron sublineages after receiving a bivalent booster dose compared with that against other Omicron sublineages (*7*) have raised concerns about potential reduction in VE against these emerging variants. Bivalent boosters contain mRNA encoding the *S-*gene from the SARS-CoV-2 ancestral strain and Omicron BA.4/BA.5 sublineages (*8*); XBB and XBB.1.5, however, are descendants of the Omicron BA.2 sublineage.^§§§^ Findings from this study suggest that bivalent booster doses are continuing to provide additional protection against symptomatic infection for at least the first 3 months after vaccination in persons who had previously received 2, 3, or 4 monovalent vaccine doses, which supports recommendations to continue to increase bivalent booster coverage.

The SGTP data in these analyses include infections with a mix of XBB, XBB.1.5, and other BA.2-related sublineages. Among specimens collected during December 1, 2022–January 2, 2023, with SGTP and with genomic sequencing results available, XBB accounted for 26%, and XBB.1.5 accounted for 33%. Together XBB/XBB.1.5 accounted for >50% of specimen sequences, which is a typical threshold considered for variant predominance in ecologic studies of variant VE (*8*). XBB.1.5, which is gaining predominance nationwide, includes one additional change in the spike receptor-binding domain compared with XBB, but it is currently unclear how this mutation might affect VE. Sensitivity analyses that assessed a subset of data from later in the analytic period when XBB.1.5 represented a larger proportion of SGTP specimens did not show differences in VE; however, because circulating variants in the United States continue to change, VE should continue to be monitored.

The findings in this report are subject to at least four limitations. First, vaccination status, previous infection history, and underlying medical conditions were self-reported and might be subject to recall bias. Self-reported frequency of previous infections differed by vaccination status, test result positivity, and SGTF status, but statistical power was not adequate to stratify by presence of previous infection >90 days earlier. Further, previous infection is likely underreported (*9*). Previous infection provides some protection against repeat infection (*10*); therefore, VE estimates in this study might be biased toward no effect. Second, bivalent booster dose coverage to date has been low (6%–39% among persons aged ≥18 years among different age groups as of January 14, 2023),^¶¶¶^ which could bias results if persons getting vaccinated earlier are systematically different from those vaccinated later. Third, data on SARS-CoV-2 exposure risk and mask use were not collected; biases might also arise because of differences in testing behaviors between vaccinated and unvaccinated persons, which might result in residual confounding. Finally, this analysis did not control for time since last monovalent dose; however, because monovalent VE against symptomatic infection with Omicron sublineages wanes quickly (*4*), this likely had a minimal effect on results.

Findings from this analysis of national pharmacy testing data show that a bivalent mRNA booster dose provided added protection against symptomatic XBB/XBB.1.5 infection for at least the first 3 months after vaccination in persons who had previously received 2, 3, or 4 monovalent vaccine doses. All persons should stay up to date with recommended COVID-19 vaccines, including receiving a bivalent booster dose when eligible.

**Summary**

**What is already known about this topic?**

The SARS-CoV-2 Omicron BA.2-related sublineage XBB.1.5 is gaining predominance nationwide. Vaccine effectiveness against XBB and XBB.1.5 is unknown.

**What is added by this report?**

Using spike (*S*)-gene target presence as a proxy for BA.2 sublineages, including XBB and XBB.1.5, during December 2022–January 2023, the results showed that a bivalent mRNA booster dose provided additional protection against symptomatic XBB/XBB.1.5 infection for at least the first 3 months after vaccination in persons who had previously received 2–4 monovalent vaccine doses.

**What are the implications for public health practice?**

As new SARS-CoV-2 variants emerge, continued vaccine effectiveness monitoring is important. All persons should stay up to date with recommend COVID-19 vaccines, including receiving a bivalent booster dose when eligible.

## Footnotes

*
https://outbreak.info

† The Social Vulnerability Index (SVI) is a tool that uses U.S. Census Bureau data on 16 social factors to rank social vulnerability by U.S. Census Bureau tract. The scale is from zero to 1; higher SVIs represent more vulnerable communities. Tests with missing SVI data (<1% of total) were excluded from all analyses. https://www.atsdr.cdc.gov/placeandhealth/svi/data_documentation_download.html

§
https://www.cdc.gov/icatt/index.html

¶ Test registrants who report receiving COVID-19 vaccines were asked to report the total number of doses and manufacturers of vaccines received and, for the most recent dose, month and year of receipt; therefore, the number of months between a vaccine dose and testing is a whole number calculated as the difference between the month and year of testing and the month and year of receipt of the vaccine dose. For doses received in the same month or the month before SARS-CoV-2 testing, an additional question was asked to specify whether the dose was received ≥2 weeks before testing; only those doses received ≥2 weeks before testing were included.

** As of January 21, 2023. Variant proportions for the most recent 3 weeks are model-based projections using the Nowcast. These projections can be uncertain or fluctuate within a wide prediction interval when a variant is just beginning to spread (i.e., has a low number of sequences and has a growth rate that is unstable). A prediction interval is an estimate of an interval in which a future observation will fall, based on what has already been observed. https://covid.cdc.gov/covid-data-tracker/#variant-proportions

†† Test registration forms asked persons to report whether they had an immunocompromising condition and provided the following examples: immunocompromising medications, solid organ or blood stem cell transplant, HIV, or other immunocompromising conditions.

§§
https://www.cdc.gov/vaccines/covid-19/clinical-considerations/covid-19-vaccines-us.html

¶¶
https://www.cdc.gov/coronavirus/2019-ncov/symptoms-testing/testing.html

*** Multivariable logistic regression models were controlled for age (adjusting for single year of age), gender, race, ethnicity, SVI of the testing location (<0.5 versus ≥0.5), underlying conditions (presence versus absence), U.S. Department of Health and Human Services region of testing location, local incidence (cases per 100,000 by individual county and state during the 7 days preceding test date), and date of testing. The following underlying conditions were included on the test registration questionnaire: heart conditions, high blood pressure, overweight or obesity, diabetes, current or former smoker, kidney failure or end stage renal disease, cirrhosis of the liver, and chronic lung disease (such as chronic obstructive pulmonary disease, moderate to severe asthma, cystic fibrosis, or pulmonary embolism).

††† 45 C.F.R. part 46.102(l)(2), 21 C.F.R. part 56; 42 U.S.C. Sect. 241(d); 5 U.S.C. Sect. 552a; 44 U.S.C. Sect. 3501 et seq.

§§§
https://www.who.int/news/item/27-10-2022-tag-ve-statement-on-omicron-sublineages-bq.1-and-xbb

¶¶¶
https://covid.cdc.gov/covid-data-tracker/#vaccination-demographics-trends
